# OTUB1 Promotes Glioblastoma Growth by Inhibiting the JAK2/STAT1 Signaling Pathway

**DOI:** 10.7150/jca.96360

**Published:** 2024-06-24

**Authors:** Jun Yang, Na Zhang, Zesong He, Junyi Xiong, Wei Meng, Chengcheng Xue, Li Ying, Meihua Li, Mei Liu, Taohui Ouyang

**Affiliations:** 1 Department of Neurosurgery, the 1 st affiliated hospital, Jiangxi Medical College, Nanchang University, No.17, Yongwai Street, Nanchang, Jiangxi province, 330006, China.; 2 Department of Neurology, the 1 st affiliated hospital, Jiangxi Medical College, Nanchang University, No.17, Yongwai Street, Nanchang, Jiangxi province, 330006, China.

**Keywords:** OTUB1, Glioblastoma, JAK2/STAT1, AZD1480, Proliferation

## Abstract

**Background:** OTUB1, an essential deubiquitinating enzyme, is upregulated in various types of cancer. Previous studies have shown that OTUB1 may be an oncogene in glioblastoma multiforme (GBM), but its specific regulatory mechanism remains unclear. This study aimed to investigate the mechanism by which OTUB1 and the JAK2/STAT1 signaling pathway co-regulate the growth of GBM.

**Methods:** Using bioinformatics, GBM tissues, and cells, we evaluated the expression and clinical significance of OTUB1 in GBM. Subsequently, we explored the regulatory mechanisms of OTUB1 on malignant behaviors in GBM *in vitro* and *in vivo*. In addition, we added the JAK2 inhibitor AZD1480 to explore the regulation of OTUB1 for JAK2/STAT1 pathway in GBM.

**Results:** We found that OTUB1 expression was upregulated in GBM. Silencing OTUB1 promotes apoptosis and cell cycle arrest at G1 phase, inhibiting cell proliferation. Moreover, OTUB1 knockdown effectively inhibited the invasion and migration of GBM cells, and the opposite phenomenon occurred with overexpression. *In vivo* experiments revealed that OTUB1 knockdown inhibited tumor growth, further emphasizing its crucial role in GBM progression. Mechanistically, we found that OTUB1 was negatively correlated with the JAK2/STAT1 pathway in GBM. The addition of the JAK2 inhibitor AZD1480 significantly reversed the effects of silencing OTUB1 on GBM.

**Conclusion:** Our study reveals a novel mechanism by which OTUB1 inhibits the JAK2/STAT1 signaling pathway. This contributes to a better understanding of OTUB1's role in GBM and provides a potential avenue for targeted therapeutic intervention.

## Introduction

Glioblastoma multiforme (GBM) is a highly aggressive primary epithelial tumor in the central nervous system, exerting the highest fatality rate among all intracranial neoplasms 1. Despite available treatments, managing GBM faces challenges such as drug resistance, tumor recurrence, and the existence of the blood-brain barrier 2, 3. Research is focusing on the molecular field for precise therapeutic targets. Targeted treatments against epidermal growth factor receptor (EGFR), interleukin (IL)-10, cluster of differentiation (CD)27, and others have been developed 4-6. However, their practical effectiveness in a clinical setting needs improvement due to the diverse functionality of these targets and the complexity of the regulatory networks involved 7. For GBM, more precise therapeutic targets or biomarkers are needed. Therefore, unraveling the molecular mechanisms of GBM pathogenesis is urgently needed.

The OTU structural domain ubiquitin aldehyde binding 1 (OTUB1) belongs to the ovarian cancer protease family, regulating ubiquitination and controlling the stability and function of proteins. Thus, it plays a crucial role in DNA damage, immune response, inflammation, apoptosis, and signal transduction 8-11. Previous studies have shown that OTUB1 may be closely related to the development of various cancers. In gastric cancer, OTUB1 exhibits a significantly heightened expression, promoting tumor proliferation through Hippo signaling activation 12. OTUB1 modulates the Wnt/β-catenin pathway in colorectal cancer to promote malignant tumor growth 13. Furthermore, OTUB1 expression was upregulated and promoted cell invasion in hepatocellular carcinoma 14. Nevertheless, understanding the regulatory mechanisms of OTUB1 in GBM is still limited, leaving its precise contribution to the initiation and progression of GBM unexplored.

In this study, we revealed that OTUB1 expression was upregulated in GBM and has a high diagnostic value. In addition, OTUB1 expression level was positively correlated with malignant behavior in GBM. We further found that OTUB1 inhibited the Janus kinase (JAK)/signal transducer and activator of transcription (STAT) signaling pathway to regulate the growth of GBM. Our study reveals a novel mechanism of OTUB1 and JAK2/STAT1 signaling pathway, emphasizing that this mechanism may have great potential for GBM therapy.

## Material and Methods

### Data mining

Sample data were analyzed by integrating the Cancer Genome Atlas (TCGA; https://portal.gdc.cancer.gov/) with the Genotype-tissue Expression (GTEx; https://commonfund.nih.gov/GTEx/) database, extracting information from UCSC XENA (https://xenabrowser.net/datapages/). Patient data obtained from the “GBM” cohort of TCGA are shown in Table S1. The extracted data were log2 (x+0.001) transformed. The results were analyzed using R software, and suitable statistics (stats and car packages) were selected based on the data format characteristics. The R package "ggplot2" was used for visualization. The R package "pROC" was utilized to calculate the Receiver Operating Characteristic (ROC) - Area Under the Curve (AUC) value of OTUB1 expression level in GBM. An AUC value exceeding 0.8 was considered reliable.

The LinkFinder module was utilized to analyze OTUB1-related genes. Pearson's correlation coefficient was used for statistical analysis. Subsequently, the LinkInterpreter module was used to carry out Gene Set Enrichment Analysis (GSEA) on OTUB1-related genes. We also performed enrichment analysis and evaluation of Kyoto Encyclopedia of Genes and Genomes (KEGG) pathways. The analyses included the establishment of ranking criteria, consideration of the false discovery rate (FDR), specification of a minimum number of genes (three), and implementation of multiple simulations (500).

### Human GBM and adjacent paired normal tissues

Twenty-four GBM samples and matched non-tumor brain tissues were acquired from the Department of Neurosurgery of the First Affiliated Hospital of Nanchang University. The patients who provided tissue samples signed an informed consent form. The protocol was approved by the Ethics Committee of First Affiliated Hospital of Nanchang University. Inclusion criteria: (1) age >20 years; (2) WHO grade IV glioma only 15. Exclusion criteria: (1) having received or currently receiving chemotherapy or radiotherapy; (2) Suffers from a disease other than GBM. The clinical parameters of the included patients are shown in Table S2.

### Cell cultures

Normal glial SVG p12 (CRL-8621) and GBM U87 (HTB-14) cells were purchased from ATCC. The T98G, U251, and U118MG cells were provided by Dr. Haibin Wu (First Affiliated Hospital of Nanchang University). All cells underwent short tandem repeat cell authentication by professional institutions. Cells were cultured in Dulbecco's modified Eagle's medium (Solarbio, Beijing, China) containing 10% serum (ExCell, Jiangsu, China) in a 37% incubator (Thermo Scientific, Waltham, USA) with an atmosphere containing 5% CO_2_.

### Cell transfection

SiRNA and the paired negative controls were purchased from Han Yi Biosciences (Guangzhou, China). Their complete siRNA sequences are detailed in Table S3. Subsequently, the siRNA with the best knockdown efficiency was selected for subsequent lentiviral construction. Lentiviral and control vectors for stable OTUB1 expression were purchased from GeneChem (Shanghai, China). The transfection process was carried out as per the manufacturer's instructions, utilizing Lipofectamine 2000 transfection reagent (Thermo Scientific, USA). Screening with puromycin (Solarbio, Beijing, China) and verification of transfection efficiency were conducted through Western blotting and reverse transcription-quantitative polymerase chain reaction (RT-qPCR).

### Western blot

Cell or sample proteins were extracted utilizing RIPA lysis buffer (Solarbio, Beijing, China). The proteins were quantified utilizing the Protein Assay Kit (Solarbio, Beijing, China). Western blotting was carried out using standard experimental procedures. Details of the antibodies are provided in Table S4.

### RT-qPCR

Total RNA was extracted using the Trizol reagent. Subsequently, mRNA was reverse-transcribed into cDNA utilizing a miRNA 1st Strand cDNA Synthesis Kit (Yeasen, Shanghai, China). Subsequently, RT-qPCR analysis was conducted on a StepOnePlus real-time PCR system (Bio-Rad, California, USA) utilizing Hieff® qPCR SYBR Green Master Mix (No Rox) (Yeasen, Shanghai, China). The primer sequences used in this study are shown in Table S5.

### Cell counting kit-8 (CCK-8) assay

The transfected cells were inoculated into 96-well plates at a density of 3000 cells per well. CCK-8 reagent (LI-COR, Shanghai, China) was added to each well at 0, 24, 48, and 72 h. Absorbance at 450 nm was measured using a zymography system after 1 h of incubation.

### Wound-healing assay

Cells were inoculated into six-well plates. Scratches were created using a 200 μL pipette tip. The images were captured with a microscope (Leica, Wetzlar, Germany) at 0 and 24 h.

### Transwell assay

Matrigel matrix gel (Corning, New York, NY, USA) was prediluted 1:8 on ice and added to the Transwell chambers. Transfected cells (6×10^5^ cells) were added to the upper chamber with 200 μL of serum-free medium. The lower chamber contained 700 μL of medium with 10% serum. The cells were incubated for 24 h for fixation and crystal violet staining. Subsequently, the chambers were photographed under an inverted microscope, and counts were obtained from five random areas.

### Cell cycle assay

Cells were washed with phosphate buffer saline (PBS) and fixed in 70% methanol for 2 h. Following another PBS wash, they were stained utilizing the Cell Cycle Detection Kit (DOJINDO, Japan) and incubated sequentially at 37 °C and 4 °C for 30 minutes each. Cells were analyzed by flow cytometry (Agilent, California, USA).

### Cell apoptosis assay

Apoptosis was investigated utilizing an Annexin V 633 Apoptosis Detection Kit (DOJINDO, Japan). After treatment, cells were twice rinsed with PBS. The working solution was added to achieve a final concentration of 1×10^6^ cells/mL in the cell suspension. Subsequently, 100 μL of cell suspension was incubated with 5 μL of annexin V 633 binding solution and 5 μL of propidium iodide solution for 15 min. Subsequently, 400 μL of the working solution was added, and the sample was analyzed within one hour using a flow cytometer (Agilent, California, USA).

### Xenograft tumor models

Four-week-old male BALB/c nude mice weighing approximately 16-18 g were purchased from GemPharmatech (Jiangsu, China). The experimental protocols were approved by the Ethics Committee of the First Affiliated Hospital of Nanchang University. Ten mice were divided into two groups of five mice each. After one week of acclimatization, 50 μL of 6 × 10^6^ U87 cells (shNC or shOTUB1) were subcutaneously injected. The volume and weight of nude mice were recorded weekly, and tumor volume was calculated using the formula (length * width^2^)/2. After four weeks, mice were euthanized, and tumors were collected and used for subsequent molecular experiments.

### Statistical methods

Statistical analyses were carried out using GraphPad Prism 9 and Microsoft Excel. Bioinformatic analyses were conducted utilizing R software version 4.2.2 (https://www.r-project.org/, accessed on February 2, 2023). Unless stated otherwise, we employed Student's t-test to evaluate and compare the disparities observed between the two groups. Differences between multiple groups were analyzed utilizing the ANOVA test. All assays were repeated at least thrice. Data are presented as mean ± standard error of the mean (S.E.M.).

## Results

### Upregulation of OTUB1 in GBM

**Figure 1:**
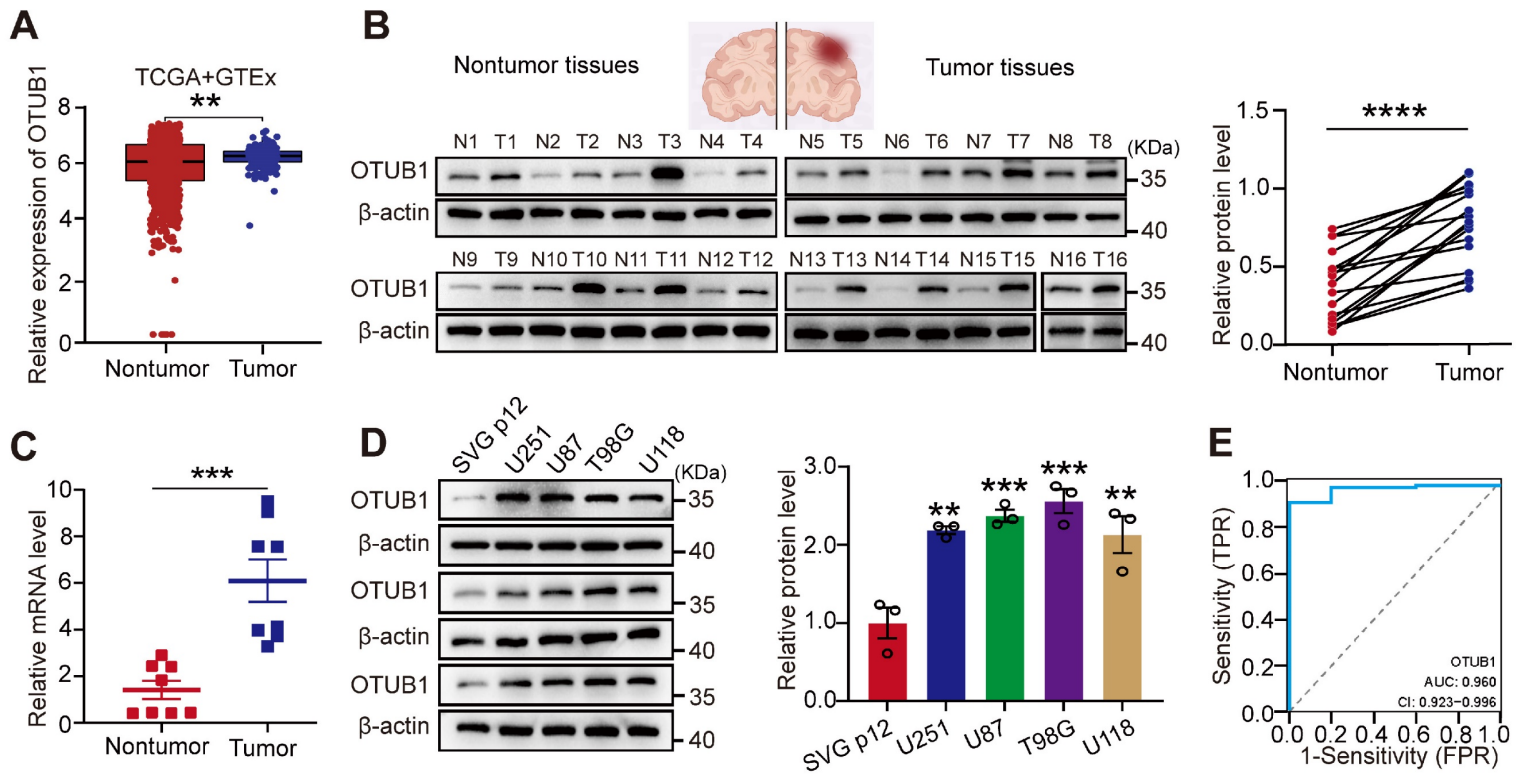
Upregulation of OTUB1 expression in GBM. (A) Comparison between OTUB1 expression in TCGA and GTEx databases for patients with glioma (166 GBM samples vs. 1157 normal brain tissue samples). (B) OTUB1 expression in GBM (n=16) and paired non-tumor tissues assessed using Western blot. (C) OTUB1 level in GBM (n=8) and paired non-tumor tissues assessed using RT-PCR. (D) OTUB1 expression in GBM and glial cells. (E) ROC curve for OTUB1 in GBM. **p<0.01, ***p<0.001, ****p<0.0001. GBM, glioblastoma; GTEx, Genotype-tissue Expression; N, nontumor; RT-PCR, reverse transcription polymerase chain reaction; ROC, receiver operating characteristic; T, tumor; TCGA, The Cancer Genome Atlas.

To investigate OTUB1 levels of expression in GBM, we analyzed TCGA and GTEx databases. As illustrated in **Figure 1A**, the expression of OTUB1 was higher in GBM tissues than in the adjacent normal tissues. The investigation of OTUB1 expression at the mRNA and protein levels using Western blot and RT-qPCR revealed that these levels of expression were higher in GBM than in adjacent non-tumor tissues (**Figure 1B and C**). Extended observations in four GBM cell types (T98G, U87, U251, and U118) revealed higher OTUB1 protein levels in GBM cells than in normal glial cells (SVG p12) (**Figure 1D).** Additionally, OTUB1 demonstrated high accuracy in GBM diagnosis as indicated by the ROC curve (AUC=0.960; **Figure 1E**). Overall, these findings underline the consistent upregulation of OTUB1 expression in GBM, warranting further investigation.

### OTUB1 knockdown inhibited metastasis and proliferation of GBM cells while promoting apoptosis and cell cycle arrest at the G1 phase *in vitro*

**Figure 2:**
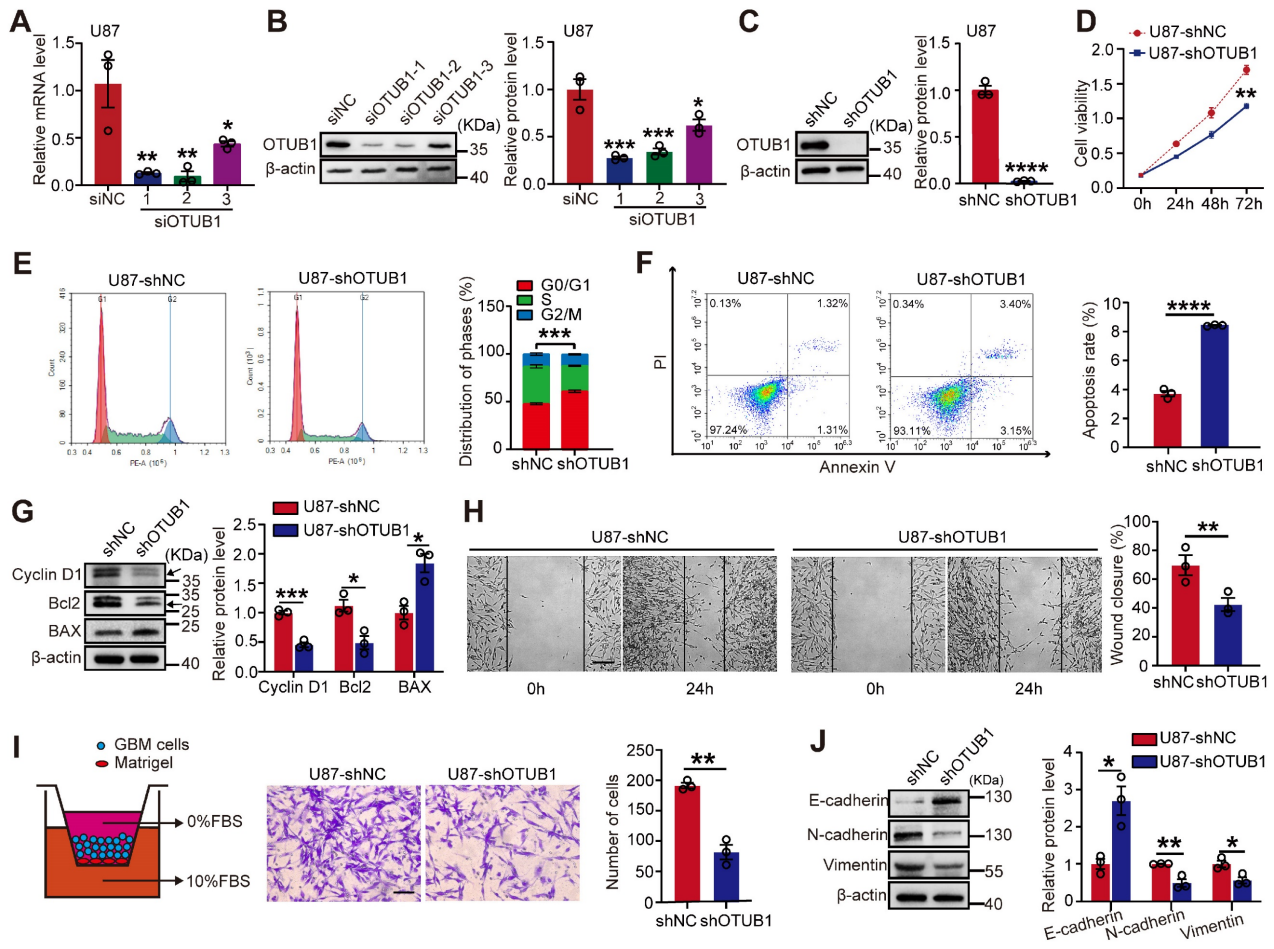
Effect of OTUB1 silencing on GBM cell metastasis and proliferation, apoptosis, and cell cycle. (A-B) The transfection efficiency of siRNAs in U87 cells assessed using Western blot and RT-PCR. (C) Stable OTUB1 protein knockdown in U87 cells assessed using Western blot. (D) The proliferation of U87 cells with OTUB1 knockdown assessed using CCK-8 assay. (E-F) Cell cycle and apoptosis of U87 cells with OTUB1 knockdown determined by flow cytometry. (G) Cell cycle- and apoptosis-related proteins in U87 cells with OTUB1 knockdown determined using Western blotting. (H-I) The migration and invasion of U87 cells with OTUB1 knockdown through wound healing and Transwell assays. Scale bar: 200μm (H), 100μm (I). (J) EMT-related protein expression after silencing OTUB1 assessed using Western blotting. *p < 0.05, **p < 0.01, ***p < 0.001, ***p < 0.0001. CCK-8, cell counting kit-8; EMT, epithelial-mesenchymal transition; GBM, glioblastoma.

To examine the impact of OTUB1 on GBM cell function, we transfected U87 cells with three siRNAs. RT-qPCR and Western blotting confirmed that siOTUB1-1 and siOTUB1-2 effectively decreased the expression of OTUB1 compared to the control (**Figure 2A and B**). Meanwhile, we constructed cells with stable OTUB1 overexpression or silencing through lentiviral transfection, verified by green fluorescent protein (GFP) fluorescence in GBM cells (**Figure S1**). As illustrated in **Figures 2C and S2A,** OTUB1 was downregulated in U87 and U251 cells.

We observed a significant reduction in cell viability in U87 and U251 cells upon silencing OTUB1, as evidenced by the CCK-8 assay (**Figures 2D and S2B**). Flow cytometry was utilized to further analyze the effect of OTUB1 on apoptosis and the cell cycle *in vitro*. Silencing OTUB1 induced G1 phase arrest and increased the proportion of apoptotic cells (**Figures 2E and F; Figures S2C and D**). Subsequently, we investigated the expressions of apoptosis- and cell cycle-related proteins. As illustrated in **Figures 2G and S2E,** the knockdown of OTUB1 decreased the expression of the cell cycle promoter cyclin D1 and the anti-apoptotic factor B-cell lymphoma 2 (Bcl2), while increasing the expression of the pro-apoptotic factor Bcl-2-associated X protein (BAX).

Sustained proliferation, high-intensity metastasis, and invasion are the main hallmarks of tumor progression 16. To assess the function of OTUB1 on the migration and invasion of GBM cells, we conducted wound healing and Transwell assays. We found that silencing OTUB1 inhibited the wound healing rate and invasive ability of U87 and U251 cells compared to controls (**Figures 2H and I; Figures S2F and G**). These results suggest that OTUB1 may be essential in GBM cell metastasis. Epithelial-mesenchymal transition (EMT) is crucial for the metastasis of cancer cells, as epithelial cells gain mesenchymal cell characteristics, enhancing cell motility and migration. OTUB1 silencing upregulated E-cadherin expressions while reducing mesenchymal cell markers N-cadherin and vimentin expressions (**Figures 2J and S2H**).

### Overexpression of OTUB1 promoted GBM cell metastasis, proliferation, G1 to S phase transition, and suppressed apoptosis *in vitro*

**Figure 3:**
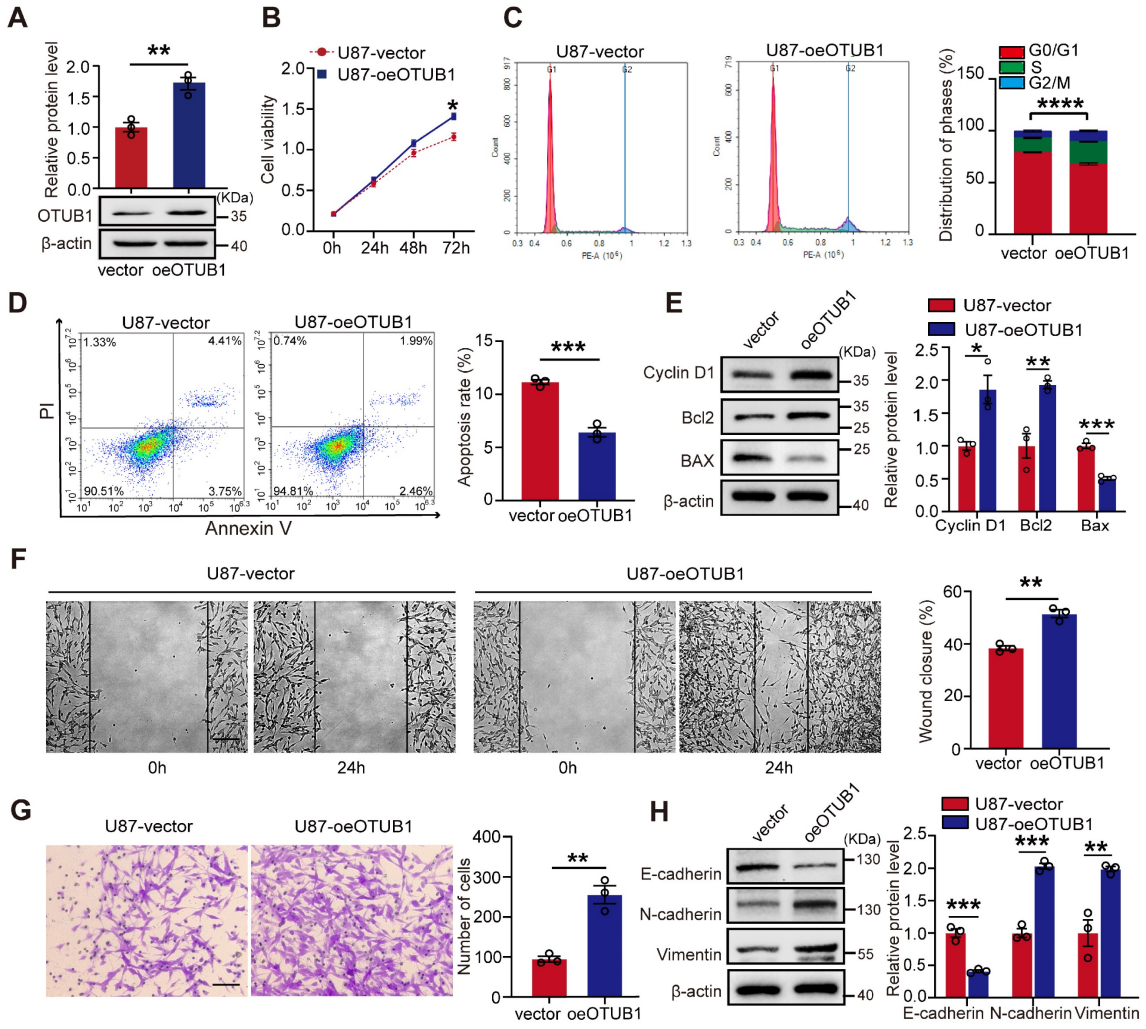
Effect of OTUB1 expression on GBM cell proliferation, migration, invasion, G1 to S phase transition, and apoptosis. (A) Stable overexpression of OTUB1 protein levels in U87 cells analyzed using Western blot. (B) Proliferation of U87 cells overexpressing OTUB1 assessed using CCK-8 assay. (C-D) Cell cycle and apoptosis in U87 cells overexpressing OTUB1 determined by flow cytometry. (E) Cell cycle- and apoptosis-related proteins in U87 cells overexpressing OTUB1 assessed using Western blotting. (F-G) The migration and invasion of U87 cells overexpressing OTUB1 evaluated using wound healing and Transwell assays. Scale bar: 200μm (F), 100μm (G). (H) Levels of EMT-related protein expression after overexpression of OTUB1 assessed using Western blotting. *p<0.05, **p<0.01, ***p<0.001, ****p<0.0001. CCK-8, cell counting kit-8; EMT, epithelial-mesenchymal transition; GBM, glioblastoma.

To further confirm the oncogenic role of OUTB1 in GBM cells, we engineered U87 and U251 cells to overexpress OTUB1 (**Figures 3A and S3A**). As illustrated in **Figures 3B and S3B,** OTUB1 overexpression promoted the viability of GBM cells. Flow cytometry analysis revealed that OTUB1 overexpression facilitated the transition of the cell cycle from G1 to S phase and concurrently inhibited apoptosis (**Figures 3C and D; Figures S3C and D**). Additionally, Western blot indicated that OTUB1 overexpression increased cyclin D1 and Bcl2 expression while reducing BAX expression (**Figures 3E and S3E**). These results strongly confirmed that OTUB1 facilitates cell proliferation and inhibiting apoptosis.

Moreover, OTUB1 overexpression enhanced the migration and invasion viability of U87 and U251 cells (**Figures 3F and G; Figures S3F and G**). Compared to control, it also significantly upregulated the expression of N-cadherin and vimentin while downregulating the expression of E-cadherin (**Figures 3H and S3H**). These findings demonstrated that OTUB1 facilitates the migration and invasion of GBM cells.

### KEGG analysis of OTUB1 co-expressed genes in GBM

**Figure 4:**
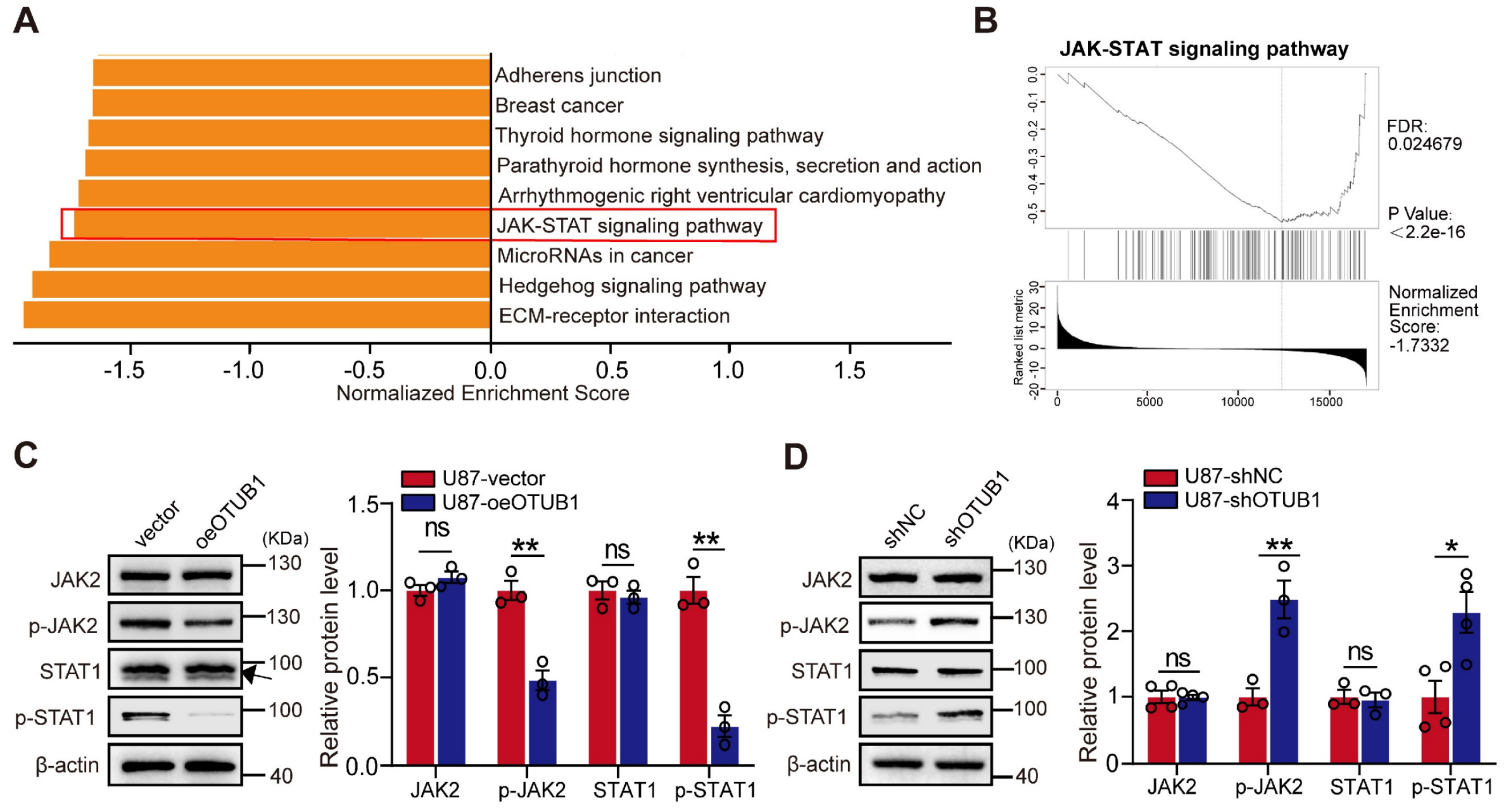
KEGG pathway analysis of genes co-expressed with OTUB1 in GBM. (A) KEGG pathway enrichment analysis of genes co-expressed with OTUB1 in GBM. (B) The correlation of genes co-expressed with OTUB1 and the JAK/STAT signaling pathway. (C-D) Expression of JAK, p-JAK2, STAT1, p-STAT1, and p-STAT1 in U87 cells with OTUB1 overexpression or silencing using Western blot. ns p>0.05, *p < 0.05, **p < 0.01. GBM, glioblastoma; GSEA, Gene Set Enrichment Analysis; Janus kinase, JAK; p-JAK, phosphorylated JAK; KEGG, Kyoto Encyclopedia of Genes and Genomes; STAT, signal transducer and activator of transcription; phosphorylated, p-STAT.

Our exploration of the involvement of OTUB1 in GBM led us to investigate its potential associations with neighboring genes in the context of GBM. We employed the LinkedOmics portal to analyze the transcriptomic data of 544 patients with GBM. We identified significant enrichment of co-expressed genes with OTUB1 in GBM, particularly within pathways such as the Hedgehog and JAK/STAT pathways (**Figure 4A**).

GSEA further unveiled a significant negative correlation between OTUB1 co-expressed genes and the JAK/STAT pathway (**Figure 4B**). We hypothesized that OTUB1 potentially inhibits the JAK/STAT1 pathway activity promoting the development of GBM. Western blot revealed that OTUB1 overexpression reduced the phosphorylation levels of JAK2 and STAT1 compared to the control groups. However, the total protein level did not change significantly (**Figures 4C and S4A**). Conversely, silencing OTUB1 resulted in a substantial increase in the phosphorylation levels of JAK2 and STAT1, while the total protein levels remained unchanged (**Figures 4D and S4B**). In summary, OTUB1 might inhibit the JAK2/STAT1 pathway in the context of GBM.

### OTUB1 inhibited the JAK2/STAT1 pathway promoting GBM cell growth

**Figure 5:**
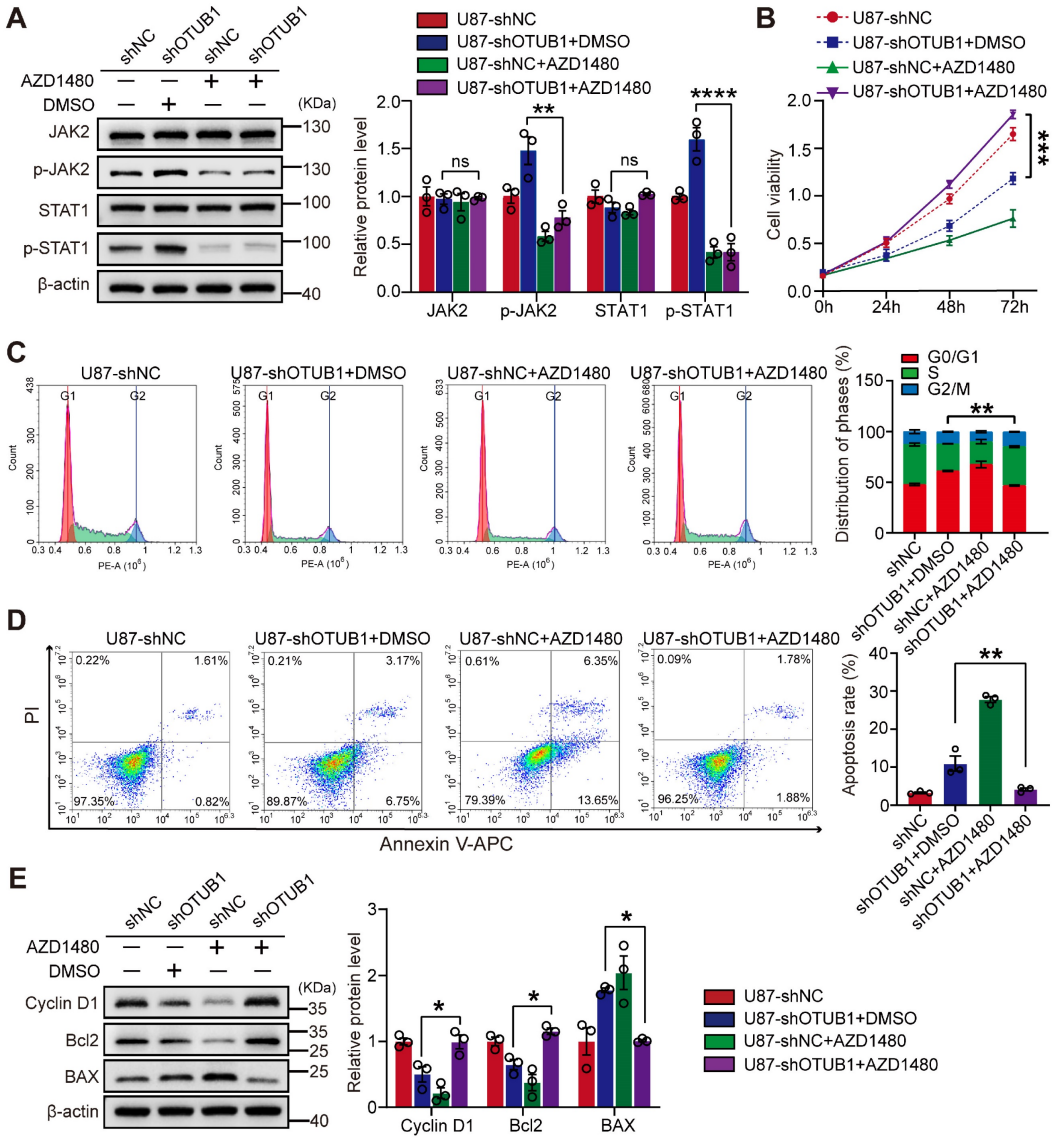
The effect of JAK2 inhibitor AZD1480 on OTUB1 silencing in GBM cells. (A) Levels of expression of p-JAK2, JAK2, p-STAT1, and STAT1 in GBM cells cultured with AZD1480 assessed using Western blot. (B) The effect of the JAK2/STAT1 pathway on the viability of OTUB1-silenced GBM cells assessed using the CCK-8 assay. (C-D) The role of the JAK2/STAT1 pathway on cell cycle and apoptosis of OTUB1-silenced GBM cells using flow cytometry. (E) BAX, Bcl-2, and cyclin D1 expression levels in GBM cells cultured with AZD1480 detected using Western blot. *p<0.05, **p<0.01, ***p<0.001, ****p<0.0001. Bcl2, B-cell lymphoma 2; BAX, pro-apoptotic factor Bcl-2-associated X protein; GBM, glioblastoma; Janus kinase, JAK; p-JAK, phosphorylated JAK; STAT, signal transducer and activator of transcription; phosphorylated, p-STAT.

To investigate whether OTUB1 can affect the cellular functions of GBM by inhibiting the JAK2/STAT1 signaling pathway, we treated the cells with the JAK2 inhibitor AZD1480 (5 μM) or the vehicle dimethyl sulfoxide (DMSO). The administration of AZD1480 effectively suppressed the phosphorylation of JAK2 and STAT1 in the context of OTUB1 knockdown (**Figure 5A**). AZD1480 reversed the effects of OTUB1 knockdown on GBM cell proliferation and invasion (**Figures 5B and S5**), cell cycle progression (**Figure 5C**), and apoptosis (**Figure 5D**). AZD1480 treatment also restored the expressions of cyclin D1, Bcl2, and BAX which were altered by OTUB1 silencing (**Figure 5E**). These findings indicated that OTUB1 inhibits the JAK2/STAT1 signaling pathway in the context of GBM, promoting cell growth and influencing various biological behaviors.

### Knockdown of OTUB1 inhibited tumor oncogenicity *in vivo*

**Figure 6:**
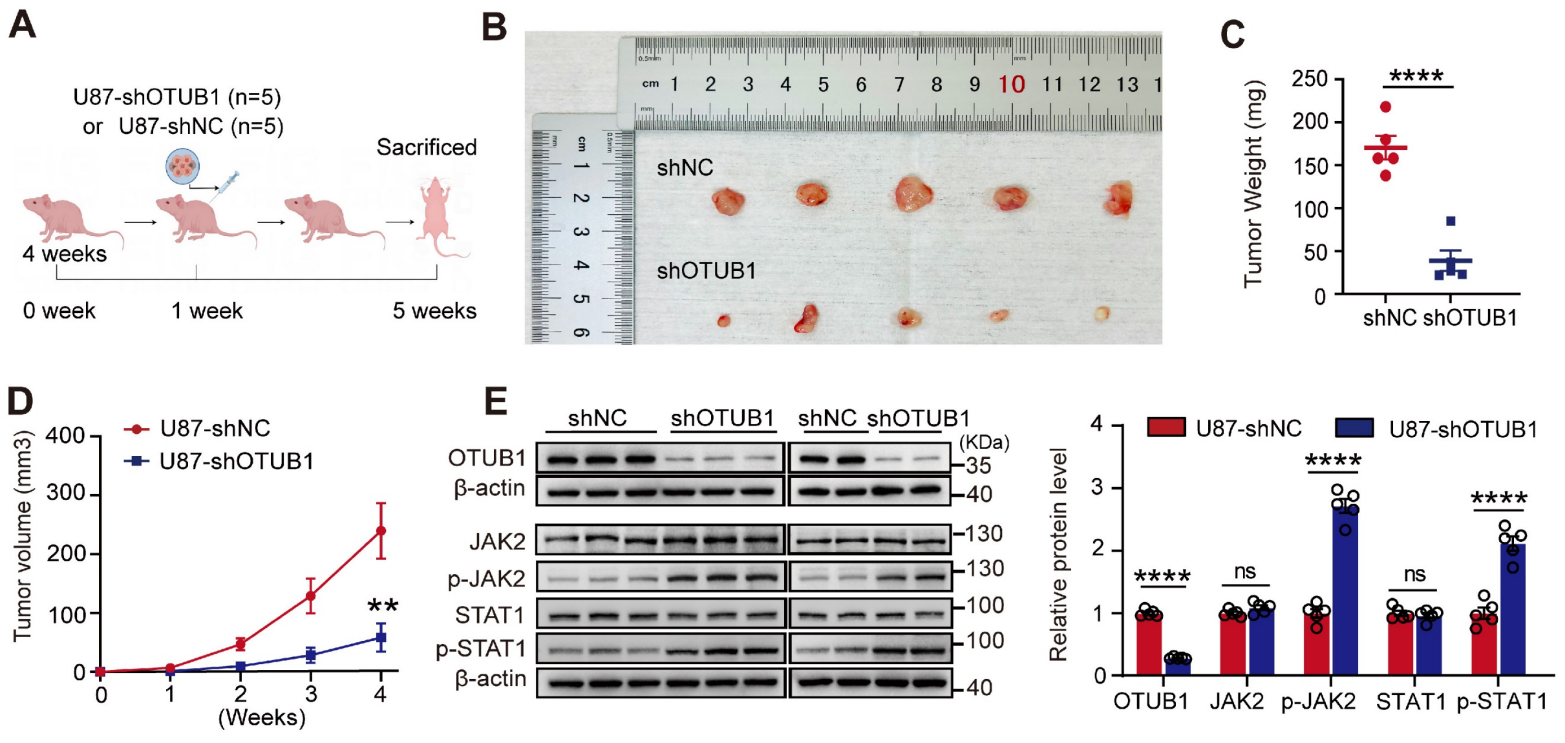
Effect of OTUB1 knockdown on tumor growth in GBM *in vivo*. (A) Flow chart outlining the subcutaneous tumor model construction in nude mice. (B) Tumor removal from shNC and shOTUB1 groups after subcutaneous U87 cell injection in nude mice. (C) Weight of harvested tumors. (D) Tumor volume changes in nude mice. (E) OTUB1 expression in extracted tumors assessed using Western blot. ns p>0.05, **p<0.01, ****p<0.0001. GBM, glioblastoma.

To validate the tumorigenic potential of OTUB1 *in vivo*, we constructed a xenograft model (**Figure 6A**). Tumors derived from cells with OTUB1 knockdown exhibited significantly slower growth than tumors formed from control-transfected cells (**Figures 6B**). As presented in **Figures 6C and D**, the OTUB1 knockdown groups exhibited a smaller tumor volume and weight compared to control groups.

Western blotting revealed that the levels of expression of OTUB1 were lower in the tumors with OTUB1 knockdown than in the tumors of the control groups (**Figure 6E**). Meanwhile, the expression of p-JAK2 and p-STAT1 was higher in the OTUB1 knockdown groups compared to the control groups (**Figure 6E**). These findings suggest that the JAK2/STAT1 pathway is activated in shOTUB1 xenograft tumors. Overall, these results underline the crucial function of OTUB1 in the oncogenicity of GBM *in vivo*.

## Discussion

OTUB1 has a dual role in tumor regulation, acting both as an oncogenic factor that facilitates tumor proliferation and metastasis but also as a potential inhibitor of tumor progression. Two studies have shown that miRNAs targeting OTUB1 impacted cancer development, indicating that OTUB1 plays a vital role in cancer cell proliferation 17, 18. Moreover, OTUB1 acts as a deubiquitinating enzyme in bladder cancer to stabilize E2F1 expression and promote malignant behavior 19. In several cancers, OTUB1 promoted cancer development through facilitating EMT 14, 20-22. In GBM, several previous studies have shown that OTUB1 may act as an oncogene, but its specific regulatory mechanisms need to be further explored.

The present study discovered a novel mechanism by which OTUB1 inhibits the JAK2/STAT1 signaling pathway. Specially, we identified OTUB1 as a GBM-specific pro-proliferative marker that inhibits the JAK2/STAT1 pathway. We analyzed the expression of OTUB1 in GBM by integrating data from TCGA and GTEx databases, revealing higher OTUB1 levels of expression in GBM tissues compared to adjacent tissues. These findings were validated using twenty-four pairs of tissues and five cell lines, aligning with the results of previous studies 22, 23. Elevated OTUB1 expression correlated with a high tumor burden, suggesting that it may promote GBM growth by inducing tumor proliferation and invasion. For comprehensive experimental validation, we constructed GBM cell lines with stable OTUB1 overexpression or silencing. The present study demonstrated that OTUB1 induced G1 to S phase transition, suppressed apoptosis, and promoted cell proliferation and invasion *in vitro*. Furthermore, we innovatively validated the oncogenicity of OTUB1 in GBM using *in vivo* xenograft models. They revealed that OTUB1 knockdown significantly inhibited tumor growth.

Furthermore, western blot analysis revealed that OTUB1 promoted GBM through facilitating EMT. We found that overexpression of OTUB1 promoted the expression of the EMT markers vimentin and N-cadherin while reducing that of E-cadherin. EMT is a transient and reversible process of cellular dedifferentiation in which cancer cells transit between different stages 24. In malignant epithelial tumors, the most aggressive subtypes typically display higher levels of EMT 25. These can be characterized by certain marker proteins, likely contributing to OTUB1 overexpression promoting GBM proliferation and invasion. Similar findings were reported by Xu *et al.*, showing that OTUB1 knockdown inhibited EMT in GBM 22.

In exploring the regulatory mechanism of OTUB1, we found that it promotes GBM growth by inhibiting the JAK2/STAT1 signaling pathway. The JAK/STAT pathway exhibits dual effects in response to various stimulatory signals, possessing both anti-tumor and pro-tumor effects depending on the variations in these signals 26. Abnormal activation of the JAK/STAT pathway is related to the development of lung adenocarcinomas 27. Conversely, the JAK/STAT signaling pathway is associated with antitumor signals in prostate and colorectal cancers 28. Furthermore, it has been shown that inhibition of the STAT1 pathway promotes glioma growth 29.

Our study revealed that OTUB1 overexpression inhibited the phosphorylation of JAK2 and STAT1, impacting GBM cell growth. Silencing OTUB1 and administering the JAK2 inhibitor AZD1480 reduced the expression of JAK2 and STAT1 phosphorylation, restoring GBM cell growth. The use of the JAK2 inhibitor significantly decreased apoptosis compared to DMSO. Therefore, we concluded that OTUB1 plays a pro-tumorigenic role in developing GBM through the JAK2/STAT1 signaling pathway. It is common for OTUB1 to act through different molecular signaling regulations. Karunarathna *et al.* demonstrated that OTUB1 inhibits ubiquitination and degradation of FOXM1, promoting epirubicin resistance in breast cancer 30. OTUB1 has been reported to directly inhibit MDM2-mediated p53 ubiquitination *in vitro* and *in vivo*, which significantly stabilizes and activates p53, causing p53-dependent apoptosis and cell proliferation inhibition phenomena 31. These suggest that OTUB1 regulates tumor physiology with multiple pathway interactions, which requires further study. Of course, our study still has some limitations. Wang *et al.* demonstrated that OTUB1 stabilizes the expression of SOCS1 through deubiquitination, inhibiting the JAK/STAT signaling pathway induced by interferon-γ 32. In oncology studies, OTUB1 can stabilize the expression of various target proteins through deubiquitination, promoting the growth of a wide range of tumors 33-35. Despite being an essential deubiquitinating enzyme, the specific molecular mechanisms underlying the involvement of OTUB1 in GBM necessitate further research to be fully elucidated. On the other hand, considering that we only included 24 GBM patients, the sample size should be expanded in future studies to analyze the association between OTUB1 expression and patients' clinical parameters.

## Conclusion

In conclusion, OTUB1 emerges as a crucial oncogene in GBM, exerting a positive regulatory effect on the biological behavior of GBM by inhibiting the JAK2/STAT1 signaling pathway. These findings suggest that targeting OTUB1 could be a promising avenue for future therapeutic approaches for GBM.

## Supplementary Material

Supplementary figures and tables.
