# Supplementary material for: OTUB1 Promotes Glioblastoma Growth by Inhibiting the JAK2/STAT1 Signaling Pathway
Source: J Cancer. 2024 Jun 24;15(14):4566–76. doi: 10.7150/jca.96360 (PMC11242346; doi:10.7150/jca.96360)
Supplement: Supplementary file 1 — Supplementary figures and tables. [file jcav15p4566s1.pdf]

OTUB1 Promotes Glioblastoma Growth By Inhibiting The JAK2/STAT1 Signaling Pathway

Jun Yang[1†]; Na Zhang [2†]; Zesong He[1]; Junyi Xiong[1]; Wei Meng[1]; Chengcheng Xue[1]; Li Ying[1]; Meihua Li[1]; Mei Liu [1*]; Taohui Ouyang[1*]

1 Department of Neurosurgery, the 1st affiliated hospital, Jiangxi Medical College, Nanchang University, No.17, Yongwai Street, Nanchang, Jiangxi province, 330006, China

2 Department of Neurology, the 1st affiliated hospital, Jiangxi Medical College, Nanchang University, No.17, Yongwai Street, Nanchang, Jiangxi province, 330006, China

* Correspondence: ndyfy05017@ncu.edu.cn (Taohui Ouyang) and ndyfy08544@ncu.edu.cn (Mei Liu)

† These authors contributed equally to this work.

Corresponding author: Correspondence: ndyfy05017@ncu.edu.cn (Taohui Ouyang) and ndyfy08544@ncu.edu.cn (Mei Liu); Institutional address: the 1st affiliated hospital, Jiangxi Medical College, Nanchang University, No.17, Yongwai Street, Nanchang, Jiangxi province, 330006, China.

Telephone: +86(0791)88694007 Fax number: +86(0791)88694007

Table S1 The clinical parameters of the patients of GBM dataset

**Characteristics**	**Low expression of OTUB1**	**High expression of OTUB1**	**P value**
n	80	81	
IDH status, n (%)			0.017
Wild-type	78 (48.4%)	71 (44.1%)	
Mutant	2 (1.2%)	10 (6.2%)	
Age, n (%)			0.134
≤60	35 (21.7%)	45 (28%)	
> 60	45 (28%)	36 (22.4%)	
Gender, n (%)			0.087
Female	33 (20.5%)	23 (14.3%)	
Male	47 (29.2%)	58 (36%)	

Note：IDH, isocitrate dehydrogenase

Table S2 The clinical parameters of the 24 GBM patients

**Characteristics**	**Number of cases**
Age (years)	
>60	7
≤60	17
Gender	
Female	12
Male	12
Tumor size (Maximum diameter)	
≥ 5 cm	15
< 5cm	9
IDH status	
Wild-type	20
Mutant	4

Note：IDH, isocitrate dehydrogenase

Table S3 Complete sequences of the three siRNAs

OTUB1-1	forward: 5′-CUACGAUAUCCUCUACAAATT-3′,<br>reverse: 5′- UUUGUAGAGGAUAUCGUAGTT-3′
OTUB1-2	forward: 5′-GCCAGGCGCUAGACAUGUATT-3′,<br>reverse: 5′- UACAUGUCUAGCGCCUGGCTT-3′
OTUB1-3	forward: 5′-GCAGGACCGAAUUCAGCAATT-3′,<br>reverse: 5′- UUGCUGAAUUCGGUCCUGCTT-3′

Table S4 Complete antibody information

Antibody name	Catalogue Number	Source of purchase	Source
OTUB1	#3783	Cell Signaling Technology	Rabbit
JAK2	#3230	Cell Signaling Technology	Rabbit
p-JAK2	#3776	Cell Signaling Technology	Rabbit
STAT1	#14994	Cell Signaling Technology	Rabbit
p-STAT1	#9167	Cell Signaling Technology	Rabbit
β-actin	66009-1-Ig	Proteintech Group	Mouse
E-cadherin	EM0502	Huabio	Mouse
N-cadherin	EM1607-37	Huabio	Rabbit
Vimentin	MA5-11883	Thermo Fisher Scientific	Mouse
Bcl2	ET1702-53	Huabio	Rabbit
BAX	ET1603-34	Huabio	Rabbit
Cyclin D1	ET1601-31	Huabio	Rabbit

Table S5 Sequences of the primers used in this study

Gene	Primer (5'–3')
OTUB1	**F:** CTGACGGCAACTGTTTCTATCG<br>**R:** CAGGTCCATGAACGTGTTGTG
β-actin	**F:** CTACCTCATGAAGATCCTCACCGA<br>**R:** TTCTCCTTAATGTCACGCACGATT

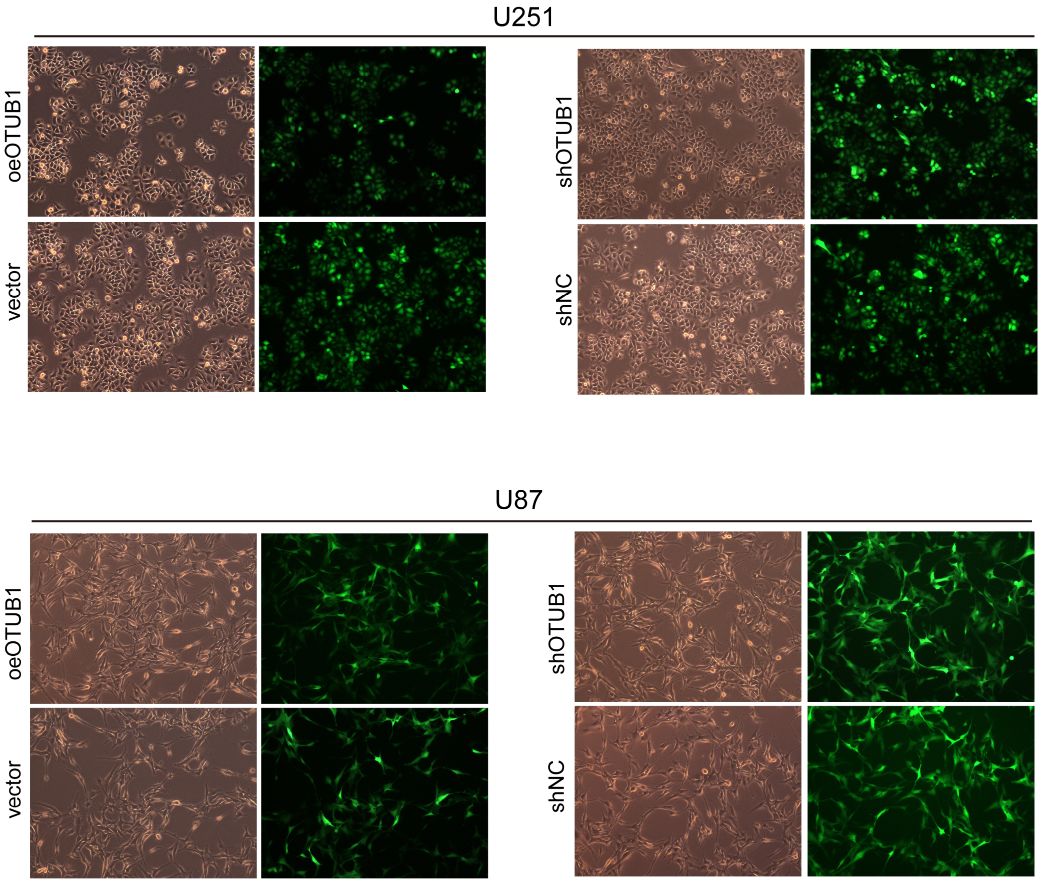

Figure S1: Green fluorescent protein (GFP) fluorescence of lentivirus overexpressing or silencing OTUB1 after transfection in U87 and U251 cells

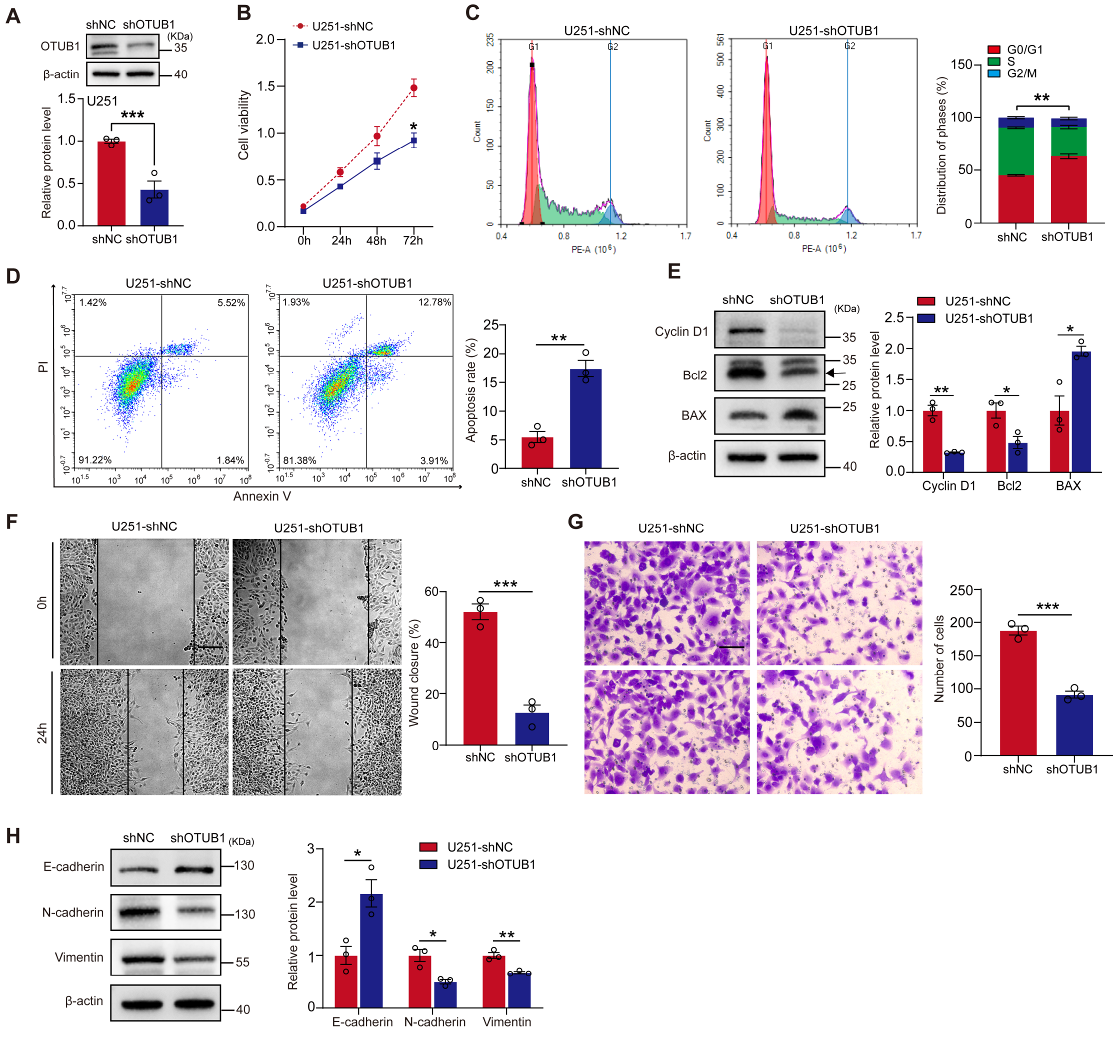

Figure S2: Silencing OTUB1 inhibited U251 cells metastasis and proliferation, promoting apoptosis and cell cycle G1 phase arrest. (A) Western blot analysis of stable knockdown of OTUB1 protein levels in U251 cells. (B) CCK-8 assay determined proliferation of knockdown OTUB1 in U251 cells. (C-D) Cell cycle and apoptosis of knockdown OTUB1 cells in U251 were determined by flow cytometry. (E) Cell cycle and apoptosis related proteins were detected in knockdown OTUB1 U251 cells by western blotting assay. (F-G) The migration and invasion of knockdown OTUB1 cells in U251 were detected by wound healing and transwell assays. Scale bar: 200μm (F), 100μm (G). (H) Western blotting analysis of EMT-related protein expression after silencing OTUB1. *$p < 0.05$, **$p < 0.01$, ***$p < 0.001$

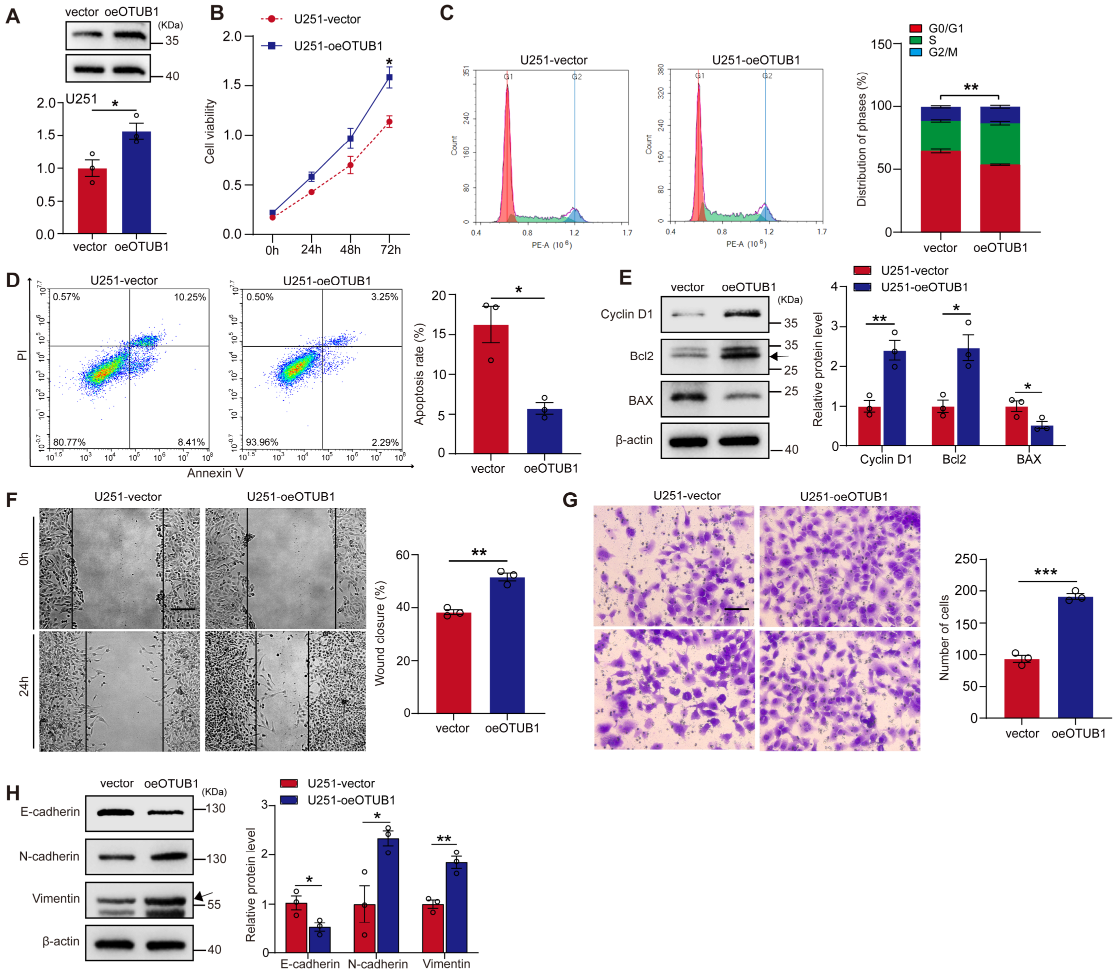

Figure S3: Overexpression of OTUB1 promotes U251 cells proliferation, migration, invasion, G1 to S phase transition, and inhibits apoptosis. (A) Western blot analysis of stable OTUB1 overexpression protein levels in U251 cells. (B) CCK-8 assay determined proliferation of OTUB1 overexpression in U251 cells. (C-D) Cell cycle and apoptosis of OTUB1 overexpression cells in U251 were determined by flow cytometry. (E) Western blotting analysis of BAX, Bcl2, and Cyclin D1 protein expression levels after overexpression of OTUB1. (F-G) The migration and invasion of overexpression OTUB1 cells in U251 were detected by wound healing and transwell assays. Scale bar: 200μm (F), 100μm (G). (H) Western blotting analysis of EMT-related protein expression levels after overexpression of OTUB1 *p<0.05, **p<0.01, ***p<0.001, ****p<0.0001

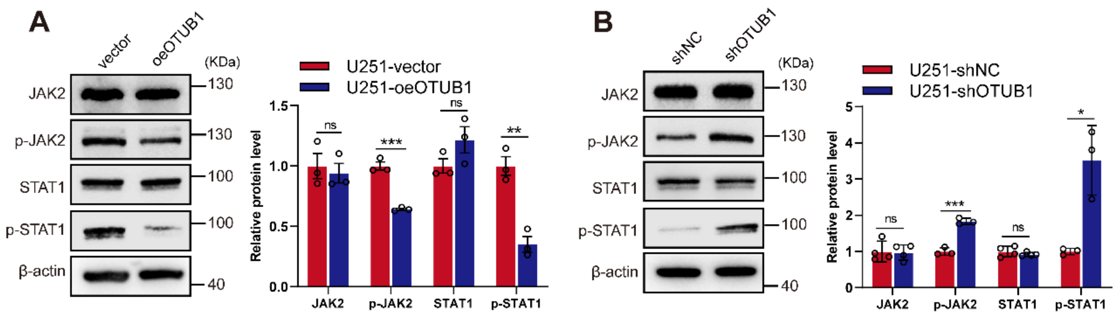

Figure S4: OTUB1 inhibits the JAK2/STAT1 signaling pathway in 251 cells. Expression of JAK, p-JAK2, STAT1, p- STAT1, and p- STAT1 was verified by western blot in U251 cells overexpressing or silencing OTUB1. ns p > 0.05, *p < 0.05, **p < 0.01, ***p<0.001

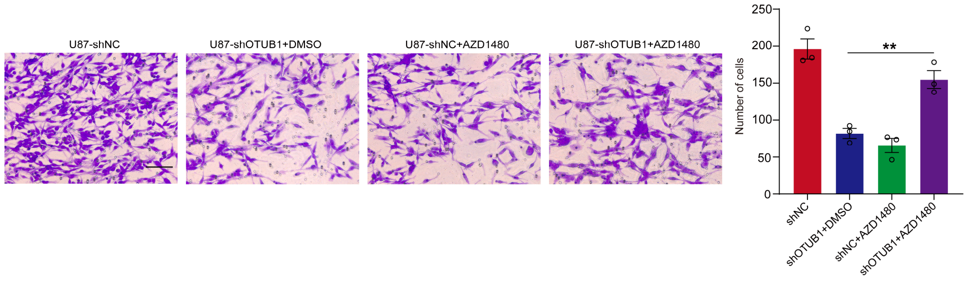

Figure S5: The JAK2 inhibitor AZD1480 reversed the effect of the knockdown of OTUB1 on GBM cells. Transwell assay demonstrated the effect of JAK2/STAT1 on cell viability in U251 cells silenced with OTUB1. ns p > 0.05, ** p < 0.01. Scale bar: 100μm
